# Phytochemical Characterization and Evaluation of Bioactive Properties of Tisanes Prepared from Promising Medicinal and Aromatic Plants

**DOI:** 10.3390/foods10020475

**Published:** 2021-02-22

**Authors:** Beatriz H. Paschoalinotto, Maria Inês Dias, José Pinela, Tânia C.S.P. Pires, Maria José Alves, Andrei Mocan, Ricardo C. Calhelha, Lillian Barros, Rafael P. Ineu, Isabel C.F.R. Ferreira

**Affiliations:** 1 Centro de Investigação de Montanha (CIMO), Instituto Politécnico de Bragança, Campus de Santa Apolónia, 5300-253 Bragança, Portugal; beatrizpaschoalinotto@hotmail.com (B.H.P.); jpinela@ipb.pt (J.P.); tania.pires@ipb.pt (T.C.S.P.P.); maria.alves@ipb.pt (M.J.A.); calhelha@ipb.pt (R.C.C.); iferreira@ipb.pt (I.C.F.R.F.); 2 Universidade Tecnológica Federal do Paraná, Campus Campo Mourão (UTFPR-CM), Campo Mourão 87301-899, Brazil; rafaelineu@utfpr.edu.br; 3 Faculty of Pharmacy, “Iuliu Hațieganu” University of Medicine and Pharmacy, 8 Victor Babeș Street, 400012 Cluj-Napoca, Romania; mocan.andrei@umfcluj.ro; 4 Laboratory of Chromatography, Institute of Advanced Horticulture Research of Transylvania, University of Agricultural Sciences and Veterinary Medicine, 400372 Cluj-Napoca, Romania

**Keywords:** medicinal and aromatic plants, herbal teas, tisane, phenolic compounds, bioactives

## Abstract

The chemical composition and biological properties correlation in several medicinal and aromatic plants is still underexplored, especially in its most common form of consumption as tisane. The present study aims to characterize the organic acids and vitamin E composition of five tisanes and their extracts by High-Performance Liquid Chromatography coupled to a diode-array detector (HPLC-DAD) and HPLC coupled to a fluorescence detector techniques, respectively, and the phenolic composition by HPLC-DAD-ESI/MS (mass spectrometry by electrospray ionization). It also focuses on their bioactive properties, namely antioxidant, antimicrobial, anti-inflammatory, cytotoxic, anti-tyrosinase, and anti-diabetic activities. A Principal Component Analysis (PCA) was performed in order to understand the correlation between the chemical composition and bioactive properties of the tisanes. The tisane 5 (T5) composed by lemon thyme, tutsan, cloves, and cinnamon, was the most promising mixture, presenting the lowest values for the lipid peroxidation inhibition, anti-inflammatory, and anti-diabetic activity. It also presented the highest concentration of phenolic acids (caffeoylquinic acids derivatives), and flavan-3-ols (catechin derivatives). Only the dry plants presented tocopherols. For the antihemolytic, antimicrobial, and cytotoxic activity, T2 and T4 (with lemon thyme) were highlighted as the best herbal mixtures. The PCA proved to be a valid tool to select the most promising tisane according to the bioactivity. These results suggest that the studied tisanes can be source of high added-value bioactive compounds with health-promoting effects and potential for application in the food and nutraceutical industries, among others.

## 1. Introduction

Currently, there is a growing interest in medicinal and aromatic plants (MAPs) due to the various benefits they provide to consumers’ health. MAPs, as natural sources of several bioactive compounds, have medicinal properties with great potential to be applied for the treatment of several diseases [1]. Their most common form of consumption is as tisane, or popularly known as herbal teas or infusions [2].

The active roles of several MAPs upon disease prevention, control, or reduction have been attributed to the antioxidant features of lypossoluble constituents (such as vitamins A and E), water-soluble components (such as vitamin C and organic acids), and phenolic compounds [3,4,5,6]. The antioxidant activity of phenolic compounds is mainly due to their redox properties, which allow them to act as reducing agents and hydrogen donators [7]. The characterization of phenolic compounds responsible for the different beneficial properties identified is of great interest for the food and nutraceutical industries, focusing on numerous applications, such as replacement of artificial antioxidants, development of food supplements, or nutraceuticals, among others [8,9]. 

There are several studies indicating that mixtures of MAPs are potentially more bioactive when compared to isolated plants [9,10]. However, the therapeutic properties that tisanes prepared from MAPs mixtures may exhibit are still under-explored. In this context, the present study describes the chemical composition in terms of phenolic compounds, organic acids, and tocopherols (vitamin E) of five tisanes prepared from mixtures of distinct MAPs. Furthermore, the antioxidant, antimicrobial, anti-inflammatory, cytotoxic, anti-tyrosinase, and antidiabetic activities of the five tisanes were also evaluated and correlated with the presence of the several families of phenolic compounds found. The results obtained could add value to these tisanes with the potential to be applied in different industrial sectors.

## 2. Materials and Methods

### 2.1. Samples and Tisanes Preparations

**Figure 1:**
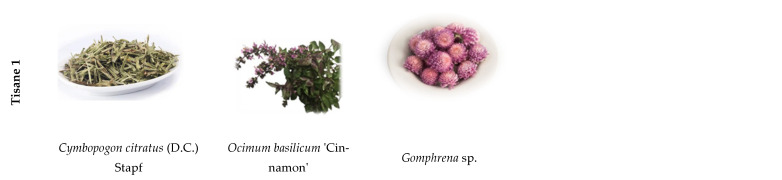

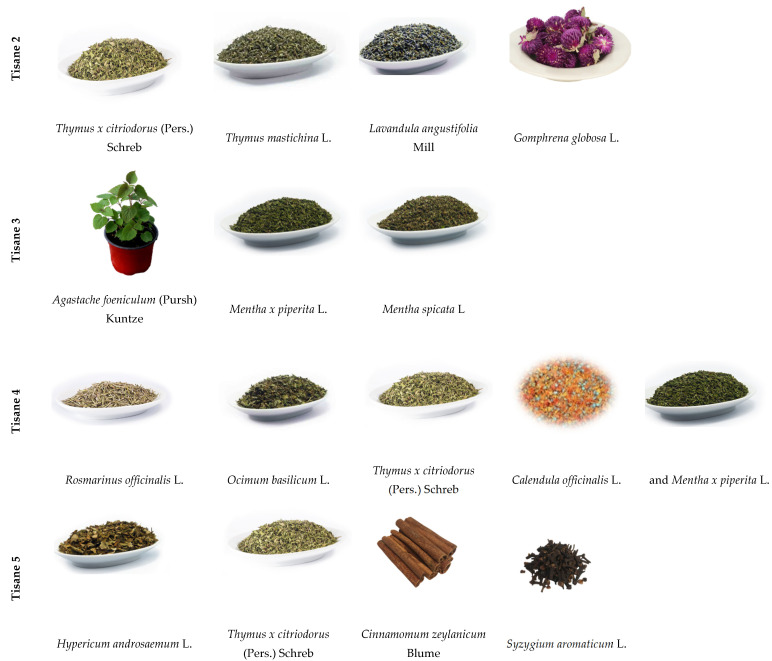
Description of the medicinal and aromatic plants (MAPs) that constituted the five samples tisanes.

Five tisane samples (Figure 1) were kindly provided by the “Cantinho das Aromáticas” Company, Canidelo—Vila Nova de Gaia, Portugal, in their commercial form (dried and packed in plastic wrappers). Tisane one (T1) was composed by *Cymbopogon citratus* (D.C.) Stapf, *Ocimum basilicum* “Cinnamon”, and *Gomphrena* sp.; tisane two (T2) had *Thymus x citriodorus* (Pers.) Schreb, *Thymus mastichina* L., *Lavandula angustifolia* Mill, and *Gomphrena globosa* L.; tisane three (T3) was composed by *Agastache foeniculum* (Pursh) Kuntze, *Mentha x piperita* L., and *Mentha spicata* L.; tisane four (T4) had *Rosmarinus officinalis* L., *Ocimum basilicum* L., *Thymus x citriodorus* (Pers.) Schreb, *Calendula officinalis* L., and *Mentha x piperita* L.; finally, tisane five (T5) was composed by *Hypericum androsaemum* L., *Thymus x citriodorus* (Pers.) Schreb, *Cinnamomum zeylanicum* Blume, and *Syzygium aromaticum* L. The MAPs that make up the tisanes were produced following the principles of organic production (certified by ECOCERT, Leiria, Portugal) and do not contain caffeine (information provided by the company). Some of the biological activities that the studied plants exhibits are described in Table 1. The dried samples were reduced to a fine homogeneous powder (~20 mesh) and stored in a cool dry place. The tisanes were prepared by adding 2 g of each dried sample to 50 mL of boiling distilled water (100 °C), allowing them to stand for 5 min at room temperature and then filtering them through a filter paper. The obtained tisanes were frozen and lyophilized, then stored in a dry place at room temperature and protected from light before further analysis. 

### 2.2. Phytochemical Characterization 

#### 2.2.1. Phenolic Compounds

The lyophilized tisane extracts were re-dissolved in water to obtain a solution of 5 mg/mL and to determine the phenolic profiles by chromatographic analysis using a Dionex Ultimate 3000 UPLC (Thermo Scientific, San Jose, CA, USA) [26]. Detection was carried out with a diode array detector (DAD) using 280 nm, 330 nm, and 370 nm as the preferred wavelengths and connected in line with a Linear Ion Trap LTQ XL mass spectrometer (Thermo Finnigan, San Jose, CA, USA) equipped with an electrospray ionization (ESI) source and working in negative mode. Data acquisition was carried out with an Xcalibur^®^ data system (Thermo Finnigan, San Jose, CA, USA). The phenolic compounds were identified through the available standard compounds and by using literature information regarding the fragmentation pattern. Quantification was performed using 5-level calibration curves obtained from available commercial standard compounds: apigenin-6-*C*-glucoside (y = 107,025x + 61,531, *R*^2^ = 0.9989, LOD = 0.19 µg/mL; LOQ = 0.63 µg/mL); apigenin-7-*O*-glucoside (y = 10,683x − 45,794, *R*^2^ = 0.996, LOD = 0.10 μg/mL; LOQ = 0.53 μg/mL); caffeic acid (y = 388,345x + 406,369, *R*^2^ = 0.9939, Limit of detection (LOD) = 0.78 μg/mL; Limit of quantification (LOQ) =1.97 μg/mL); catequin (y = 84,950x – 23,200, *R*^2^ = 0.999, LOD = 0.17 μg/mL; LOQ = 0.68 μg/mL); chlorogenic acid (y = 168,823x − 161,172, *R*^2^ = 0.9999, LOD = 0.20 µg/mL; LOQ = 0.68 µg/mL); cinnamic acid (y = 1 × 10^6^x − 222,204, *R*^2^ = 0.9993, LOD = 0.33 µg/mL and LOQ = 1.19 µg/mL); ferulic acid (y = 633,126x − 185,462, *R*^2^ = 0.999, LOD = 0.20 μg/mL; 1.01 μg/mL); naringenin (y = 18,433x + 78,903, *R*^2^ = 0.9998, LOD = 0.17 µg/mL; LOQ = 0.81 µg/mL); *p*-coumaric acid (y = 301,950x + 6966.7, *R*^2^ = 0.9999, LOD = 0.68 μg/mL and LOQ = 1.61 μg/mL); quercetin-3-*O*-glucoside (y = 34,843x − 160,173, *R*^2^ = 0.9998, LOD = 0.21 µg/mL; LOQ = 0.71 µg/mL); quercetin-3-*O*-rutinoside (y = 13,343x + 76,751, *R*^2^ = 0.9998, LOD = 0.14 µg/mL; LOQ = 0.45 µg/mL); rosmarinic acid (y = 191,291x − 652,903, *R*^2^ = 0.999, LOD = 0.15 µg/mL; LOQ = 0.68 µg/mL); and syringic acid (y = 376,056x + 141,329, *R*^2^ = 0.9995, LOD = 0.23 µg/mL and LOQ = 0.72 µg/mL). The results were expressed in mg/g of extract. 

#### 2.2.2. Organic Acids and Tocopherols (Vitamin E) 

The organic acids were analyzed by ultra-fast liquid chromatography coupled to a photodiode array detector programmed to record at 215 nm as the preferred wavelength (UFLC-PDA; Shimadzu Coperation, Kyoto, Japan) [27]. The identification and quantification of the individual organic acids was performed by comparison to authentic standards and by comparison of the peak area in the programmed wavelength. Standard calibration curves for organic acids quantification: oxalic acid (y = 9 × 10^6^x + 45,973, *R*^2^ = 0.9901, LOD = 12.55 µg/mL; LOQ = 41.82 µg/mL); quinic acid (y = 610,607x + 46,061, *R*^2^ = 0.9995, LOD = 24.18 µg/mL; LOQ = 80.61 µg/mL); malic acid (y = 912,441x + 92,665, *R*^2^ = 0.999, LOD = 35.76 µg/mL; LOQ = 119.18 µg/mL); and fumaric acid (y = 154,862x + 1 × 10^6^, *R*^2^ = 0.9977, LOD = 0.08 μg/mL; LOQ = 0.26 μg/mL). The results were expressed in g per 100 g of dry weight for dry plants and in mg per 100 mL of infusion preparations.

Tocopherols were determined using HPLC coupled to a fluorescence detector (FP-2020; Jasco, Easton, MD, USA) programmed for excitation at 290 nm and emission at 330 nm [12]. The identification was performed by chromatographic comparisons with authentic standards, while the quantification was based on the fluorescence signal response of each standard, using the internal standard (tocol) method and by using calibration curves obtained from commercial standards of each compound. The results were expressed in g per 100 g of dry weight for dry plants and in mg per 100 mL for infusion preparations.

### 2.3. Evaluation of Bioactive Properties

#### 2.3.1. Antioxidant Activity

The antioxidant activity was evaluated through the lipid peroxidation inhibition using porcine brain cell homogenates by using the thiobarbituric acid reactive substances (TBARS) assay [28] and oxidative hemolysis inhibition assay (OxHLIA) [29]. The lyophilized tisane extracts were re-dissolved in water to obtain stock solution of 0.3 mg/mL, which were further diluted to obtain a range of seven concentrations below the stock solution for the TBARS assay. For OxHLIA, the lyophilized tisane extracts were re-dissolved in PBS, while water was used for complete hemolysis and PBS solution as the control. The results were expressed as IC_50_ values (µg/mL), with the sample concentration providing 50% of lipid peroxidation inhibition or extract concentration required to keep 50% of the erythrocyte population intact for Δ*t* of 120 and 180 min, respectively. Trolox was used as a positive control.

#### 2.3.2. Antimicrobial Activity

The lyophilized tisane extracts were re-dissolved in water to obtain a stock solution of 20 mg/mL, while the samples were serially diluted obtain the concentration ranges (20 at 0.15 mg/mL). The antimicrobial activity was evaluated using five Gram-negative bacteria: *Escherichia coli*, *Proteus mirabilis*, *Klebsiella pneumoniae*, *Pseudomonas aeruginosa,* and *Morganella morganii*, and three Gram-positive bacteria: *Enterococcus faecalis*, *Listeria monocytogenes*, and methicillin-resistant *Staphylococcus aureus* (MRSA). The Minimum Inhibitory Concentration (MIC) and the Minimum Bactericidal Concentration (MBC) were performed using the colorimetric assay of *p*- iodonitrotetrazolium [30]. Three negative controls were prepared (one with Mueller Hinton Broth/ Tryptic Soy Broth (MHB)/(TSB), another one with the extract, and the third with medium, antibiotic, and bacteria). One positive control was prepared with MHB/TSB and each inoculum. Ampicillin and Imipenem were used for all Gram-negative bacteria tested and *Listeria monocytogenes*. Ampicillin and vancomycin were selected for *Enterococcus faecalis* and MRSA.

#### 2.3.3. Anti-Inflammatory Activity

The anti-inflammatory potential was evaluated by producing nitric oxide formed in the mouse macrophage-like cell line RAW 264. 7 [28]. The lyophilized tisane extracts were re-dissolved in water to obtain stock solutions of 8 mg/mL, which were further diluted to obtain a range of six concentrations below the stock solution. For the determination of nitric oxide, a Griess Reagent System kit was used, which contains sulphanilamide, *N*-(1-napthyl)ethylenediamine hydrochloride (NED), and nitrite solutions. A reference curve of nitrite (sodium nitrite 100 mM to 1.6 mM; y = 0.0063x + 0.1368; *R*^2^ = 0.9989) was prepared in a 96-well plate. The nitric oxide produced was determined by measuring the absorbance at 540 nm (microplate reader ELX800 Biotek), and by comparison with the standard calibration curve. The results were expressed in IC_50_ values (µg/mL), which correspond to the sample concentration providing 50% of inhibition of nitric oxide (NO) production. Dexamethasone was used as a positive control. 

#### 2.3.4. Cytotoxic and Hepatotoxic Activity

The lyophilized tisane extracts were re-dissolved in water to obtain stock solution of 8 mg/mL, which were further diluted to obtain a range of six concentrations below the stock solution. HepG2 (hepatocellular carcinoma), NCI-H460 (non-small cell lung carcinoma), HeLa (cervical carcinoma), and MCF-7 (breast adenocarcinoma) were used as human tumor cell lines. Non-tumor cells were also tested; a cell culture (named as PLP2) was prepared from a freshly harvested porcine liver obtained from a local slaughterhouse, according to a procedure established by the authors. Sulforhodamine B assay was performed according to a procedure previously described by the authors [28]. Ellipticine was used as a positive control and the results were expressed in GI_50_ values (µg/mL), which correspond to the sample concentration providing 50% of inhibition of cell growth. 

#### 2.3.5. Anti-Tyrosinase Activity

The tyrosinase inhibitory activity of each lyophilized herbal tea extract was determined using the SPECTROstar Nano Multi-Detection Microplate Reader and 96-well plates (BMG Labtech, Ortenberg, Germany) [31]. Absorbance was measured at 475 nm, and results are expressed as IC_50_ or percentage of inhibition (when the sample is not active enough to calculate an IC_50_ value for the tested concentration of 8 mg/mL) using the following calculation formula: % I = ((AB) − (CD))/((AB)) × 100. Kojic acid solution (0.10 mg/mL) was used as a positive control.

#### 2.3.6. Antidiabetic Activity 

The capacity of inhibition α-glucosidase (from yeast and rat) was measured in a 96-well microplate reader [32,33]. The lyophilized tisane extracts were re-dissolved in water to obtain a stock solution of 8 mg/mL. Acarbose was used as a positive control. The results were expressed as IC_50_ values (µg/mL) or percentages of inhibition (when the sample is not active enough to calculate an IC_50_ value for the tested concentration of 8 mg/mL) using the following calculation formula: Inhibition (%) = ((Abscontrol − Absort)/Abscontrol) × 100.

### 2.4. Statistical Analysis

For each extract and its infusion, all the assays were carried out in triplicate. The results were expressed as mean values and standard deviation (SD) and analyzed using one-way analysis of variance (ANOVA) followed by Tukey’s HSD test with *p* = 0.05 (except for the analysis of anti-inflammatory activity results, in which a Student’s *t*-test was used, with α = 0.05) using IBM SPSS Statistics for Windows, Version 23.0. (IBM Corp., Armonk, NY, USA). For the PCA analysis, a categorical principal component analysis (CATPCA) with optimal scaling was used to explore the joint relationships between the five tisanes in study and their phenolic composition and bioactive capacity. The number of plotted dimensions (two) was chosen in order to allow meaningful interpretations and was assessed by the respective eigenvalues (should be greater than one), the Cronbach’s alpha (must be positive) and the total percentage of variance (should be as higher as possible) explained by the selected data.

## 3. Results and Discussion

### 3.1. Phenolic Compounds 

The five tisanes presented a very distinct phenolic compounds profile (Table 2). However, it was possible to group the identified phenolic compounds into five different families: phenolic acids, isoflavones, flavanones, flavonoids, and flavan-3-ols, and quantify them accordingly, as is shown in Table 3. 

Twenty-nine phenolic acids (caftaric acid, syringic, coutaric, *p*-coumaric, fertaric, caffeic, chicoric, rosmarinic acids, and their derivatives), 21 flavonoids (*C*-glycosylated apigenin derivatives and *O*-glycosylated kaempherol, luteolin, quercetin, isorhamnetin, chrysoeriol, apigenin, and diosmetin derivatives), four isoflavones (*O*-glycosylated calycosin and derivatives), three flavanones (*O*-glycosylated eriodictyol derivatives), two flava-3-ols (B-type (epi)catechin trimer and tetramer), and three non-phenolic compounds (medioresinol derivatives and oleanolic acid derivative), were tentatively identified in the tisanes samples studied. Two unknown compounds were also found in tisanes T3 and T5. 

As previously stated, the tentative identification of the phenolic and non-phenolic compounds was performed comparing the DAD-MS results with available commercial standards and by comparison with data reported in the literature. Table 2 provides a description of the methodology used for the identification of each compound in which bibliographic references report the chromatographic data of the compound in question in detail, for valid identification. An effort was also performed to try to reconcile the references with the individual plant matrices present in each sample; however, it was not possible to achieve this for many of the compounds. The bibliographic references that have plant matrices in common to those studied in the present work were Miguel et al. [50] and Pires et al. [38], who studied the flowers of marigold; Rita et al. [51] and Roriz et al. [52] presented the phenolic profile of lemongrass, lemon thyme [51], and *G. globosa* [52]; Pereira et al. [53] studied the phenolic profile of pepper mint and Ribeiro et al. [45] studied the phenolic profile of rosemary. All of these bibliographic references were also used for identification of many of the phenolic compounds reported herein. 

Regarding the concentrations (mg/g of extract) of the various families of compounds in each tisane (Table 3), it was found with regard to flavonoids, the vast majority of compounds were glycosylated derivatives of luteolin (peaks 21, 31, 34, 35, 44, 46, and 52), preceded by glycosylated quercetin derivatives (peaks 25, 26, 30, 33, and 41), except for T3 samples. The highest concentration of glycosylated derivatives of luteolin and quercetin were found in T2/T4 samples (peak 34) and T2 (24), respectively. The glycosylated derivatives of kaempherol were only identified in tisane T2 (peak 20, kaempherol-*O*-diglucuronide) and in trace amounts. Regarding the glycosylated derivative of isorhamnetin, it was exclusively identified in T4 sample (peak 28, isorhamnetin-*O*-di-deoxyhexosyl-hexoside). There are several references regarding the bioactive properties of this family of phenolic compounds, the most relevant of which are its anti-inflammatory, anti-tumor, and antioxidant properties [54].

The glycosylated derivatives of eriodictyol were the only flavanones tentatively identified in the tisanes samples (except in the T1 sample, where were not flavanones identified). The highest concentration of total flavanones was found for T4 samples (7.876 mg/g of extract, due to the presence of the peaks 24 and 27) as shown in Table 3. 

### 3.2. Organic Acids and Tocopherols

The results for the characterization of organic acids and tocopherols in mixtures of dried tisanes (extracts) and infusions preparations are shown in Table 4.

The content and composition in organic acids and tocopherols vary according to the typology of different plant tissues, since the production of these compounds depends on the edaphoclimatic conditions of plant growth, namely light, salinity, and temperature [46,53]. Regarding the organic acid content, it was noted that the composition is very similar in all tisane samples, both in dry extracts and in infusion preparations, with oxalic, quinic, malic, and fumaric acids being identified. However, T1 samples (dry extract and infusion preparation) presented the highest concentrations of these compounds (4.59 g/100 g dry weight and 234 mg/100 mL, respectively), mainly due to the presence of oxalic acid. Sample T5 presented the lowest levels of organic acids, all of them in the trace form. Fumaric acid presented the lowest expression of all identified organic acids and was only detected in the T5 tisane. 

Regarding the tocopherol composition (Table 3), as expected, these molecules were identified only in the dry extracts, and no isoforms were detected in the infusion preparations. These molecules are fat soluble and their extraction with water is inefficient, having a low affinity with this type of extraction solvent [55]. Therefore, it can be stated that tisanes from extracts of the MAP mixtures studied are not sources of tocopherols (vitamin E). Concerning the dry extracts, only two isoforms (α and γ) were detected, with the γ-tocopherol isoform being the most outstanding. The total content of tocopherols varied significantly among all tisane samples, with T1 extract presenting a higher amount of α-tocopherol (12.2 mg/100 g of dry weight), and T5 extract presenting a higher amount of γ-tocopherol (109 mg/100 g dry weight), which significantly affected its total content. Consequently, T5 tisane revealed the highest concentration of total tocopherols (112 mg/100 g dry weight).

In *C. Citratus*, oxalic, malic, ascorbic, succinic, and fumaric acids have already been described by Roriz [10], but in the tisane where this plant is present, ascorbic, succinic and fumaric acids have not been detected, possibly due to competitiveness in the face of the other two plants that are present in T1 samples (*O. basilicum* “Cinnamon” and *Gomphrena* sp.). Oxalic and malic acids have also been previously described in *Gomphrena* sp. by Liberal et al. [15].

Oxalic acid was the compound identified in the vast majority of aqueous extracts and preparations (except for T4 tisane extract and T3 and T4 aqueous preparation). This organic acid has the ability to inhibit the lipase enzyme and, consequently, to reduce the occurrence of physiological problems such as type II diabetes [56]. On the other hand, quinic acid is considered as a powerful antioxidant, having a stronger performance than the synthetic antioxidant BHT (butylated hydroxyl toluene) [57]. Regarding malic acid, it participates in the Krebs cycle and therefore has an essential role in human metabolism, having also been shown to have a bactericidal effect [58,59]. The use of organic acids in the food industry is a sustainable and natural alternative and can be used as a preservative, antioxidant, flavoring and acidifier [58], and their presence in tisanes may confer bioactive properties on a daily intake basis.

Vitamin E is composed of four isoforms (α—alpha, β—beta, γ—gamma, and δ—delta), and its most biologically active isoform is α-tocopherol, therefore the most necessary in the human diet. The isoform α is responsible for 50% of the attributed antioxidant action, while the isoform β has 25–50% and the isoforms γ and δ has between 10–35% [60].

Two of the plants that make up the T1 tisane have already been studied individually regarding their composition in tocopherols. In 2014, Roriz [10] reported the presence of the isoforms α and γ tocopherol in *C. citratus*, with α-tocopherol being the isoform present in the highest concentration. In turn, Liberal et al. [15] identified the same isoforms for *Gomphrena* sp., with γ-tocopherol being the most abundant isoform. On the third plant that makes up the T1 tisane (*O. basilicum* “Cinnamon”), there is no data regarding the composition in tocopherols. In the study carried out by Roriz [10], antagonistic effects were described in mixtures of MAPs as being responsible for differentiating the content in bioactive compounds, namely tocopherols and organic acids, depending on the concentration of the plants. This may explain the differences in relation to the *C. citratus* plant, since its concentration in T1 tisane may possibly be insignificant and therefore the same composition and content in tocopherols and organic acids was not obtained when compared with the literature.

Regarding the T5 tisane, Ramadan, Asker, and Tadros [60] identified the four isoforms of tocopherol (α, β, γ and δ) in the essential oil of *Syzygium aromaticum* (cloves) in expressive quantities, with the isoform α- the predominant. In 2015, Gandomani et al. [61] reported an increase in the levels of α-tocopherol in egg yolk when supplementary powder from the sprout of the *S. aromaticum* plant was added, thus indicating the major contribution of the isoform in this species. For *H. androsaemum*, it was not possible to find published data related to the content in tocopherols. However, Hosni, Msaâda, Taârit, and Marzouk [62] reported the isoforms α, γ, and δ-tocopherol in four species belonging to the genus *Hypericum* and identified δ-tocopherol as the most abundant isoform, although its amount presented great variations depending on the species. It was also not possible to find data regarding the content of tocopherols in *T. citrodorus*; however, this plant also belongs to the order Lamiales, which predominantly presents the isoforms α and γ, as previously mentioned. According to the national reference of food composition, the National Institute Dr. Ricardo Jorge [63], cinnamon (*Cinnamomum zeylanicum*) has 0.1 g of total tocopherol for every 100 g of edible part. Thus, the dried extracts from the tisanes can be considered as sources of this type of bioactive compounds and, therefore, can be used in various applications, namely nutraceuticals.

### 3.3. Evaluation of Bioactive Properties

#### 3.3.1. Antioxidant Activity 

The results of the antioxidant activity assays are described in Table 5 and are expressed as IC_50_ (μg/mL). In the OxHLIA assay, IC_50_ values were given at two-time intervals (Δ*t*), since natural extracts contain different antioxidant molecules capable of interacting with each other and favoring the protection of cell membranes at different time periods. The five tisanes samples tested showed antioxidant capacity for both assays performed. All showed significant differences, except T3 and T4 tisanes for the TBARS assay, probably because they have a common plant (*Mentha x piperita* L.) in the composition of their respective extracts that contributed to this activity, as described by Pereira et al. [53]. For the OxHLIA assay, the tisane that presented the best antioxidant activity, and consequently the best antihemolytic action, was the T2 sample, at 120 and 180 min, with an IC_50_ of 17.8 μg/mL and 30.3 μg/mL, respectively. This result suggests that the compounds present in tisane T2 can reduce hemolytic action by 50% for 120 min and extend for an additional 60 min. For TBARS, the most active tisane was the T5 sample with an IC_50_ of 5.07 μg/mL, that is the minimum concentration capable of inhibiting 50% of lipid peroxidation. In both assays performed, the lowest activity was shown by the T1 sample. 

The IC_50_ obtained for both assays was lower than the standards used, as shown in Table 5, making these tisanes promising sources of compounds with antioxidant capacity. Similar results were observed for herbal tea plants that have common plants, such as tisanes T2, T5, and T3 with lemon thyme (*T. citriodorus*). 

In the OxHLIA assay, the antioxidant activity was found in the following order: T2 > T3 > T5 in 120 min and T2 > T5 > T3 in 180 min. The inversion of bioactivity presented between T3 and T5 for the times of 120 and 180 min is probably due to the fact that these two herbal teas have different antioxidant molecules depending on the other plants that make up the mixture of herbal teas (in T3 with anise and mint, and in T5 with St. John’s wort, cinnamon and cloves). In the TBARS test, the results of antioxidant activity were ordered as follows: T5 > T2 > T3 = T4, since T3 and T4 have one plant in common (peppermint). 

The mechanisms of antioxidant activity related to cells are believed to be through the capture of free radicals and the alteration of cell membrane properties, often due to phenolic compounds, in particular, flavonoids. Some of these compounds can be incorporated into the hydrophobic core of the membrane bilayer, causing a reduction in the fluidity and stability of the membrane. The reduction in fluidity can limit the diffusion of free radicals and improve the antioxidant efficacy of flavonoids; however, how the solubility of these compounds affects the stability of the cell membrane remains uncertain [64].

Therefore, the conviction of the antioxidant activity of plant matrices related to the composition and quantity of phenolic compounds is reinforced. The presence of phenolic acids confers the ability to inhibit lipid peroxidation in pig brain tissues (TBARS), as they exhibit a high antioxidant activity, being grouped into benzoic acids (such as synergic acid, *p*-hydroxybenzoic acid, and gallic acids) and cinnamic acids (as an example: *p*-coumaric, ferulic, and caffeic acids), and it can also bond with each other or with other compounds to form potential structures, such as chlorogenic acid (a combination of caffeic acid and quinic acid). The antioxidant potential of phenolic compounds is strongly correlated with the number of hydroxyl groups attached to the aromatic rings. In addition, hydroxyl groups in the *ortho* and *para* position increase the anti-oxidative and anti-radical activity of phenolic acids [64].

#### 3.3.2. Antimicrobial Activity 

Data on antimicrobial activity based on minimum inhibitory concentrations (MIC) and minimum bactericidal concentrations (MBC) are shown in Table 5. The highest antimicrobial potential for Gram-positive bacteria was showed by tisane T3 (values of 5 to 10 mg/mL for minimal inhibition), except for methicillin-resistant *Staphylococcus aureus* (MRSA) for which the most active herbal tea was sample T2. In general, tisane T2 was the only one that had a minimum inhibitory concentration of 20 mg/mL for Gram-negative bacteria, while for the other tisanes (T1, T3, T4, and T5) higher concentrations would have required 20 mg/mL to inhibit the growth of this type of bacteria. The tisane T5 was found to be the lowest potential tisane because it exhibited the highest MIC and MBC values. All tisanes (T1, T2, T3, T4, and T5) inhibits the bacterial growth of some of the bacteria tested, but none has bactericidal activity.

The antimicrobial activity of aqueous extracts is promising, since organic extracts may present cytotoxicity to the human organism. However, the concentration required for the activity presented by the herbal tea tested in the present study is still relatively high when compared to the same organic extracts [45,46]. 

Cushnie and Lamb [65] described the antimicrobial capacity of flavonols/flavones (apigenin, naringenin, luteoline, isoflavones, flavanones, quercetin, and their glycosylated derivatives), as these types of molecules show activity against several bacterial strains. This statement agrees with the results obtained in the present work, since the T3 tisane sample presented higher antibacterial capacity for Gram-positive bacteria and, in turn, showed a higher concentration of flavonols/flavones.

#### 3.3.3. Anti-Inflammatory Activity 

The results for the anti-inflammatory activity are shown in Table 5. Only two tisanes showed anti-inflammatory action, namely T5 and T4, and the first showed higher activity with an IC_50_ of 345 μg/mL. The other three tisanes (T1, T2, and T3) presented concentrations above 400 μg/mL, not revealing anti-inflammatory properties. 

It was already described in the literature that the individual plants that make up the tisanes mixtures have anti-inflammatory activity, such as *C. citratus* [10], *G. globosa* [15,66], *T. mastichina* [67], *Mentha x piperita* [19], *R. officinalis* [23,68], *O. basilicum* [20], *C. officinalis* [12], *H. androsaemum* [16], and *Syzygium aromaticum* [69]. However, in most cases extracts are obtained using organic solvents.

Jabeur et al. [16] reported the inhibition of nitric oxide production by *H. androsaemum* hydroethanolic extract (plant present in tisane T5), indicating an anti-inflammatory action for the same trial performed in the present study and it was correlated with the significant amount of chlorogenic acid derivatives present in the extract. These results are in agreement with the results obtained in this study, since in T5 the *trans* forms of neochlorogenic acid (3-*O*-caffeoylquinic acid), cis and trans cryptochlorogenic (4-*O*-caffeoylquinic), and cis and trans forms of chlorogenic acid (5-*O*-caffeoylquinic) were significantly quantified.

There are other compounds present in tisane T5, which are also attributed to the bioactive property in question, such as luteolin. According to Sung and Lee [70], this flavonoid also exhibits anti-inflammatory properties by suppressing nitric oxide production. Regarding the T4 sample, apigenin, luteolin, coumaric acid, and quercetin were identified as potentially anti-inflammatory compounds, which were referred to above. Although samples T2 and T3 contain some of these compounds as chlorogenic acid, luteolin, and apigenin, these tisanes were not promising sources of anti-inflammatory activity, possibly due to the occurrence of synergistic reactions between the various compounds identified and the low concentration of the compounds referred to as anti-inflammatory potentials when compared to T4 and T5. Therefore, the MAP mixtures present in T4 and T5 have positive effects against inflammatory diseases, although relatively high concentrations are required.

#### 3.3.4. Cytotoxic and Hepatotoxic Activity 

The results for the four human tumor cell lines and primary culture of non-tumor pig cells are shown in Table 5. The highest cytotoxic capacity was observed for lung carcinoma tumor cell line (NCl H460). The T4 sample was the tisane with the highest cytotoxic potential, as the lowest GI_50_ values were observed for three of the four tumor cell lines tested: lung carcinoma (248 μg/mL), cervical adenocarcinoma (HeLa, 253 μg/mL), and hepatocellular carcinoma (HepG2, 264 μg/mL), respectively. For the MCF-7 (human breast adenocarcinoma) cell line, the most cytotoxic tisane was T5 with GI50 = 285 μg/mL. The tisane T2 did not show cytotoxic potential for any of the tumor cell lines tested, although it has in its composition lemon thyme (*T. citriodorus*), which is also present in T4 and T5 extracts, thus reinforcing the important need for further studies on the synergistic and antagonistic effects on mixtures of MAPs in tisanes preparations marketed. The hepatotoxicity was also evaluated for all the samples (Table 4), and a concentration higher than 400 µg/mL was observed in all tisanes, which means that no sample revealed toxicity against PLP2 cells.

The literature describes the cytotoxicity of the individual plants present in the tisane T4. Pereira et al. [53] observed promising effects in the methanolic extract of peppermint (*M. piperita*) for the same tumor cell lines. For the marigold sample (*C. officinalis*), it was described by Miguel et al. [50] that this shows cytotoxic activity for the same lines with values greater than 250 μg/mL. The aqueous extract of rosemary did not show antiproliferative effects against tumor cell lines when studied by Gonçalves et al. [71], exposing that the anticancer effects attributed to this plant come from diterpenes [72], which were not identified in the tisane T4. However, the cytotoxic capacity shown by this herbal tea may be related to the other plants belonging to the mixture.

#### 3.3.5. Anti-Tyrosinase Activity 

The results of the anti-tyrosinase activity assay based on the inhibition of the tyrosinase enzyme from mushrooms are described in Table 5. The results were expressed as a percentage of inhibition of the enzyme action at a maximum concentration of 8 mg/mL (maximum concentration tested). All samples of herbal teas (T1, T2, T3, T4, and T5) did not show inhibition capacity of the tyrosinase enzyme at the maximum tested concentration of 8 mg/mL.

Tisanes are not able to specifically inhibit this enzyme from mushrooms. Tyrosinase is an enzyme that contains copper and is widely distributed in microorganisms, animals and plants. Tyrosinase from mushrooms has now become popular because it is widely available [73] and is able to inhibit the formation of neuromelanin associated with the development of Parkinson’s (PD) [74]. The inhibition of this enzyme is one of the main strategies for the treatment of hyperpigmentation, since several dermatological disorders, such as melasma, age spots, and actinic damage, result from the accumulation of an excessive level of epidermal pigmentation, and, therefore, compounds tyrosinase inhibitors have become increasingly important to be incorporated into cosmetic products [72,73,74]. Currently, new sources are sought, preferably natural ones, which are capable of inhibiting tyrosinase, since the inhibitor compounds most commonly used by the industry have many limitations, such as low activity and high cytotoxicity [75].

#### 3.3.6. Antidiabetic Activity 

Data on antidiabetic activity based on inhibition of α-glucosidase of fungal and animal origin are shown in Table 5. The α-glucosidase inhibitors are an important class of antidiabetic drugs that prevent the sudden increase in hyperglycemia after meals [76]. The tisanes T1 and T3 did not present a minimum inhibition concentration of 50% of fungal α-glucosidase, therefore, the percentage inhibition was calculated as previously mentioned and demonstrated the ability to inhibit the action of α-glucosidase by 41% and 43%, respectively. The tisane T5 showed the best antidiabetic activity for fungal origin and the only one that presented satisfactory concentration for inhibition of 50% of the enzyme of animal origin. The tisanes T1, T2, T3, and T4 were not sufficiently active for animal activity at the maximum tested concentration of 8 mg/mL. 

No data were found for antidiabetic activity for the plants that make up the tisane T5; however, Adisakwattana et al. [77] described the inhibitory activity of potent α-glucosidase for *Orthosiphon aristatus* belonging to the Lamiaceae family, which corresponds to the same family of *T. citriodorus* (lemon thyme) present in tisane T5. Among polyphenols, chlorogenic acid has received much attention for several biological activities including excellent inhibition of α-amylase and type II diabetes-linked α-glucosidase [78]. This is in accordance with the present study, since in the T5 sample, the most abundant compounds are chlorogenic acids (CGA) and derivatives.

### 3.4. Selecting the Most Promisssing Tisane

**Figure 2:**
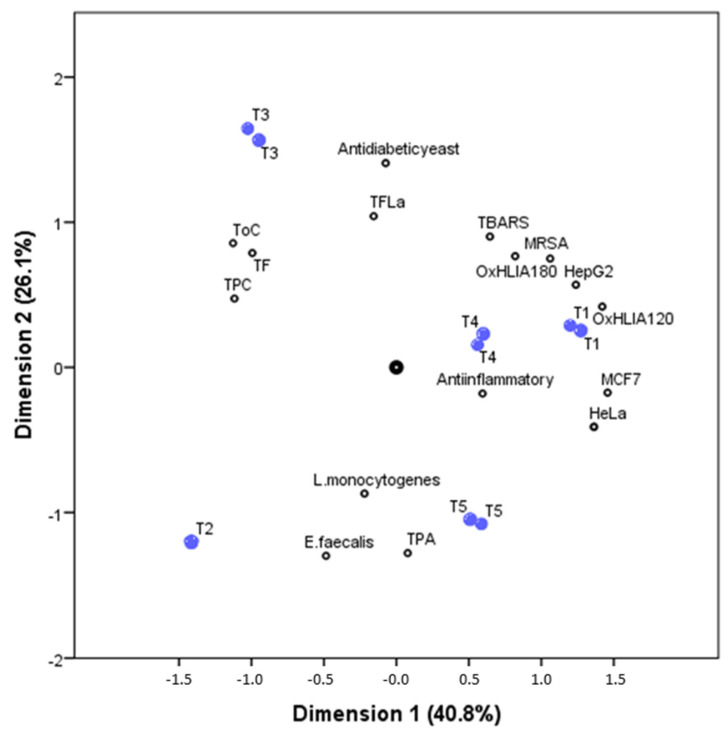
Biplot of object scores (plant tisanes, blue dots) and component loadings (families of phenolic compounds and some bioactive properties, black dots) from CATPCA. The first two dimensions (first: Cronbach’s α, 0.917; eigenvalue, 7.308; second: Cronbach’s α, 0.864; eigenvalue, 5.359) account for most of the variance.

A principal component analysis was performed in order to better understand the correlations between the phenolic and non-phenolic compounds composition with the bioactive properties in the five studied tisanes samples. The biplot illustrated by Figure 2, joints the relations between precisely the five tisanes (object scores) and their phenolic composition and bioactive properties (component loadings). The first two-dimensions (first: Cronbach’s α, 0.917; eigenvalue, 7.308; second: Cronbach’s α, 0.864; eigenvalue, 5.359) account for most of the total variance (40.8% and 26.1%, respectively).

The first dimension was effective in separating the tisanes samples while taking into account their content in phenolic and non-phenolic compounds; the second dimension was effective in separating the tisanes by their antioxidant and cytotoxic activity, but was particularly effective in the separation of samples by their antidiabetic yeast activity and antimicrobial action against *L. monocytogenes* and *E. faecalis*. 

T3 sample is positively correlated with the family of compounds TF (total flavonoids), ToC (total other compounds), and TPC (total phenolic compounds), meaning that this sample present the highest concentrations, while being negatively correlated with the bacterial strains *L. monocytogenes* and *E. faecalis* (presenting the lowest MIC values, and for that manner the highest antibacterial activity against this strains). TPA (total phenolic acids) is strongly correlated with the T5 sample and is moderately correlated with T2 samples; these two samples’ negative correlation with the vast majority of the bioactive activities studied means lower IC_50_, GI_50_, and MIC values, and therefore indicates higher bioactive potential. 

## 4. Conclusions

Comparatively the phenolic composition of tisanes and phenolic acids were the family of compounds that stood out the most (primarily in the T5 sample), due to the presence of chlorogenic acid and rosmarinic acid and their derivatives, both qualitative and quantitative (with the exception of the T3 sample in which there were higher levels of flavonoids). In turn, the family of flavonoids also stood out in all of them, due to the presence of glycosylated derivatives of apigenin, kaempherol, quercetin, luteolin, diosmetin, and calycosin. It was also possible to identify flavanones (glycosylated derivatives of eriodictyol), flavan-3-ols (derived from (epi)catechin) and still other types of no-phenolic compounds (two lignans and a pentacyclc triterpenoid). 

With the study of the composition in organic acids and tocopherols, it was found that oxalic acid had higher levels for all samples and it is the T1 sample that revealed the highest total content of organic acids in the dry extract and aqueous preparation. Regarding tocopherols, it was found that in the aqueous preparation of tisanes, no isoform was detected due to the lipid character of this molecule and γ-tocopherol was the most abundant isoform in the dry extract, mainly in T5 sample.

Regarding the results obtained for the various bioactivities studied, it is again the T5 tisane sample that stands out, revealing the highest capacity for the inhibition of lipid peroxidation in the TBARS assay, better cytotoxic potential against the MCF-7 cell line, higher anti-inflammatory activity, and anti-diabetic activity with the inhibition of α-glucosidase of fungal and animal origin (having been the only one to reveal activity for the enzyme of animal origin). For the oxidative hemolysis inhibition test, the T2 sample stands out, revealing the lowest IC_50_ values for 120 and 180 min. However, the T4 sample is the one that revealed the greatest cytotoxic potential for three cell lines, since it presented lower GI_50_ values. Regarding antimicrobial activity, the T3 sample showed the lowest MIC values for Gram-positive bacteria. It should be noted that no sample revealed hepatotoxicity against the non-tumor line of pig liver cells. Furthermore, none of the samples showed anti-tyrosinase activity at the maximum tested concentration of 8 mg/mL and for Gram-negative bacteria, the vast majority tisanes did not show very promising results, as they presented MIC and MBC results higher than the maximum tested concentration (20 mg/mL).

The presence of lemon thyme in several of the mixtures of MAPs in tisanes may enhance the most promising results for bioactive activities, since it is present in tisanes with the best evaluated biological properties. The three tisanes in which the lemon thyme makes up the mixture are T2, T4, and T5, which are precisely those that stand out with the highest biological properties. The correlations presented by the Principal Component Analysis (PCA) reinforce the correlation between the presence of phenolic and non-phenolic compounds in the five tisanes and its bioactivities. 

The antioxidant action of polyphenols presents in tisanes has sparked interest in the food industry as possible natural substitutes for current artificial antioxidants. In addition to their recognized bioactive potential, they also have the ability to inhibit oxidative changes that occur in food. Several groups of polyphenols (anthocyanins, flavanones, and isoflavones) are currently used in industry as components of nutraceuticals and functional foods. The interest within the pharmaceutical industry is to find natural products obtained from plants in relation to the current artificial compounds available for the treatment of different health problems. The development of resistance to antimicrobials, the reappearance of infectious diseases, high production costs, and limited useful life of therapeutic agents are important factors that have encouraged interest in drugs that include derivatives of plant extracts.

Studies like the one proposed with this work are extremely important to chemically characterize the plants commonly used in traditional medicine, adding value to them as sources of molecules that can later be applied in a wide range of industrial sectors.

## Figures and Tables

**Table 1:** Applicability, common uses, and biological properties of the MAPs that constituted the five samples tisanes.

Plant	Family	Applicability/Common Uses	Bioactive Properties	Ref.
*Agastache foeniculum* (Pursh) Kuntze	Lamiaceae	Medicine	Antimicrobial	[11]
*Calendula officinalis* L.	Asteraceae	Cosmetic, medicinal, food, and colorant	Anti-inflammatory, antitumor, and antimicrobial	[12]
*Cinnamomum zeylanicum* Blume	Lauraceae	Food	Antitumor and antidiabetics	[13]
*Cymbopogon citratus* (D.C.) Stapf	Poaceae	Cosmetic, pharmaceutical, and infusion	Antioxidant, intestinal anti-inflammatory, antitumor, and antidiabetics	[10]
*Gomphrena globosa* L.	Amaranthaceae	Infusion	Antioxidant, anti-inflammatory, and antidiabetics	[14]
*Gomphrena* sp.	Amaranthaceae	Bioactive and infusion	Antioxidant, antimicrobial, and antitumor	[15]
*Hypericum androsaemum* L.	Hypericaceae	Medicine	Anti-inflammatory, antitumor, antimicrobial, antioxidant, and antiviral	[16]
*Lavandula angustifolia* Mill.	Lamiaceae	Perfumery, cosmetics, and medicine	Antimicrobial, antioxidant, antiviral, and cytotoxic	[17]
*Mentha spicata* L.	Lamiaceae	Food, pharmaceutical, perfumery, confectionery	Antioxidant, antimicrobial, and neuroprotective effects	[18]
*Mentha x piperita* L.	Lamiaceae	Food, infusion	Antioxidant, antimicrobial, and anti-inflammatory	[19]
*Ocimum basilicum* L.	Lamiaceae	Food	Anti-inflammatory, antimicrobial, and neuroprotective effects	[20]
*Ocimum basilicum "Cinnamon”*	Labiatae	Flavoring agent and essential oil	Antioxidant and antimicrobial	[21,22]
*Rosmarinus officinalis* L.	Lamiaceae	Culinary and medicine	Antioxidant, antimicrobial, anti-inflammatory, anti-tumor, and anti-diabetic	[23]
*Syzygium aromaticum* L.	Myrtaceae	Medicine	Anti-inflammatory, antioxidant, antimicrobial, and anti-tumor	[23]
*Thymus mastichina* L.	Lamiaceae	Food, cosmetics, and infusion	Antioxidant, anti-inflammatory, antitumor, and antifungal	[23]
*Thymus x citriodorus* (Pers.) Schreb	Lamiaceae	Food, infusion	Antimicrobial, anti-inflammatory, and antioxidant	[24,25]

**Table 2:** Retention time (Rt), wavelengths of maximum absorption in the visible region (λ_max_), mass spectral data, and tentative identification of phenolic and non-phenolic compounds found in the tisanes prepared from five MAP mixtures (T1, T2, T3, T4, and T5).

Peak	Rt (min)	λmax (nm)	[M-H]^−^ (*m*/*z*)	MS^2^ (*m*/*z*)	Tentative Identification	Reference/Method Used for Identification
1	4.75	263/325	311	179(72),149(100),135(5)	Caftaric acid	[33]
2	4.79	324	353	191(100),179(49),173(10),135(12)	3-*O*-Caffeoylquinic acid	DAD/MS [34,35]
3	5.5	280	359	197(100),153(8),113(5)	Syringic acid hexoside	[36]
4	5.81	312	295	163(100),149(5),119(5)	Coutaric acid	[33]
5	6.31	264	325	163(100),119(18)	*p*-Coumaric acid hexoside	[37]
6	6.62	324	353	191(80),179(9),173(100),135(5)	*cis* 4-*O*-Caffeoylquinic acid	[34,35]
7	6.67	290/321	325	193(100),149(5),134(5)	Fertaric acid	[33]
8	7.06	285	611	449(100),287(13)	Eriodictyol-*O*-dihexoside	[33]
9	7.11	324	353	191(100),179(5),173(5),135(5)	*trans* 4-*O*-Caffeoylquinic acid	[34,35]
10	7.87	325	353	191(100),179(9),173(5),135(5)	*cis* 5-*O*-Caffeoylquinic acid	[34,35]
11	8.41	281	387	369(25),207(100),163(41),119(5)	Medioresinol	[38,39]
12	8.54	321	353	191(100),179(12),173(5),135(5)	*trans* 5-*O*-Caffeoylquinic acid	[34,35]
13	9.95	325	593	473(100),383(13),353(21),325(5)	Apigenin-*C*-hexoside-*C*-hexoside	[40]
14	10.05	322	179	135(100)	Caffeic acid	DAD/MS
15	11.1	281	865	739(83),713(50),695(100),577(77),425(12),407(10),289(5),287(12)	Type B (epi)-catechin trimer	[41]
16	11.44	279	325	163(100),145(5),119(8)	*p*-Coumaric acid hexoside	[37]
17	12.12	283	1153	865(37),577(12),561(5),289(5),287(5)	Type B (epi)-catechin tetramer	[41]
18	12.22	327	473	311(100),293(98),179(61),149(80),135(5)	*cis* Chicoric acid	[42]
19	12.3	327	473	311(100),293(95),179(50),149(55),135(5)	*trans* Chicoric acid	[42]
20	12.59	324	637	461(100),285(15)	Kaempherol-*O*-diglucuronide	DAD/MS
21	13.46	346	637	285(100)	Luteolin-*O*-diglucuronide	DAD/MS
22	14.47	330	555	537(100),493(12),359(10),179(5),161(5)	Salvianolic acid K isomer	[43]
23	14.5	285/325	537	493(100),359(34),313(13),295(4),269(3),197(5),179(5)	Lithospermic acid A	[44]
24	15.16	284	595	287(100)	Eriodictyol-*O*-deoxyhexosyl-hexoside	[45]
25	15.18	341	477	301(100)	Quercetin-*O*-glucuronide	[46]
26	15.8	344	463	301(100)	Quercetin-3-*O*-glucoside	DAD/MS
27	15.99	282	463	287(100)	Eriodictyol-*O*-glucuronide	DAD/MS
28	16.96	342	769	315(100)	Isorhamnetin-*O*-di-deoxyhexosyl-hexoside	[46]
29	17.37	322	521	359(100),197(45),179(40),161(15),135(5)	Rosmarinic acid hexoside	[44]
30	17.69	343	609	301(100)	Quercetin-3-*O*-rutinoside	DAD/MS
31	17.98	345	593	285(100)	Luteolin-*O*-deoxyhexosyl-hexoside	[45]
32	18.02	346	593	285(100)	Luteolin-7-*O*-rutinoside	DAD/MS
33	18.07	348	477	301(100)	Quercetin-*O*-glucuronide	[46]
34	18.38	347	461	285(100)	Luteolin-7-*O*-glucoside	DAD/MS
35	18.93	347	461	285(100)	Luteolin-*O*-glucuronide	[46]
36	18.57	280	717	519(100),475(20),339(7),321(5)	Salvianolic acid B	[44]
37	18.96	281/327	487	325(90),307(51),293(100),193(5),179(12)	Feruloylcaffeoyl tartaric acid	[38]
38	19.2	347	521	359(100),197(44),179(38),161(14),135(5)	Rosmarinic acid hexoside	[44]
39	19.29	282/323	719	539(24),521(17),339(100),197(10),179(5),161(5),135(5)	Sagerinic acid	[44]
40	19.85	337	717	537(40),519(100),493(5),359(10),339(5),321(5),295(5),197(5),179(5)	Salvianolic acid B isomer	[44]
41	20.17	340	549	505(100),301(10)	Quercetin-*O*-malonylhexoside	DAD/MS
42	21.92	330	359	197(29),179(36),161(100),135(5)	Rosmarinic acid	DAD/MS
43	22.25	325	555	537(5),511(8),493(45),311(100),197(5),179(5),161(5),135(5)	Salvianolic acid K	[43]
44	23.92	340	533	489(100),285(10)	Luteolin-*O*-malonylhexoside	DAD/MS
45	24.05	341	549	387(100),369(21),207(50),163(41),119(5)	Medioresinol caffeate	[38,39]
46	24.71	333	461	285(100)	Luteolin-*O*-glucuronide	[46]
47	25.51	335	475	299(100),285(5)	methylluteolin-*O*-hexuronide (chrysoeriol hexuronide)	[47]
48	25.85	331	717	537(10),519(100),493(5),359(5),339(21),321(50),295(9),197(5),179(5)	Salvianolic acid B isomer	[44]
49	25.97	324	537	493(100),359(10),313(6),295(6),269(5),197(5),179(5)	Lithospermic acid A	[44]
50	28.17	330	517	473(20),269(100)	Apigenin-*O*-malonylhexoside	DAD/MS
51	29.02	331	503	285(100)	Luteolin-*O*-malonylpentoside	DAD/MS
52	29.71	341	547	503(50),299(100)	Diosmetin-*O*-malonylhexoside	DAD/MS
53	30.17	284/329	537	493(100),439(51),197(24),179(6),359(20)	Lithospermic acid A isomer 2	[44]
54	30.48	280/339	493	359(100),313(15),295(9),269(7),197(5),179(5)	Salvianolic acid A	[45]
55	30.84	330	503	285(100)	Luteolin-*O*-malonylpentoside	DAD/MS
56	33	268/331	677	659(21),451(100),367(10),225(20)	Unkwown	-
57	34.19	290	791	773(100),678(11),546(2),451(35),337(2),225(11)	Oleanolic acid derivative	[48]
58	35.77	268/333	1063	771(100),283(50),269(10)	Unkwown	-
59	36.87	338	589	545(100),299(5)	Diosmetin-malonyl methyl hexoside	DAD/MS
60	38.08	269/329	283	269(100)	Calycosin	[49]
61	40.01	269/329	573	283(100)	Calycosin derivative	DAD/MS
62	40.36	268/329	533	283(100)	Calycosin derivative	DAD/MS
63	41.83	268/330	573	283(100)	Calycosin derivative	DAD/MS

**Table 3:** Quantification (mg/g of extract) of the phenolic and non-phenolic compounds present in tisanes prepared from five MAP mixtures (mean ± SD).

Peak	T1	T2	T3	T4	T5	*t*-Students Test *p*-Value
1 ^A^	0.41 ± 0.01	nd	nd	nd	nd	-
2 ^B^	nd	nd	nd	nd	3.13 ± 0.14	-
3 ^C^	nd	0.163 ± 0.002	nd	nd	nd	-
4 ^D^	0.143 ± 0.001	nd	nd	0.151 ± 0.002	nd	0.001
5 ^E^	nd	0.863 ± 0.01	nd	0.29 ± 0.01	nd	<0.001
6 ^B^	nd	nd	0.69 ± 0.02	nd	0.63 ± 0.03	<0.001
7 ^F^	0.172 ± 0.002	nd	nd	nd	nd	-
8 ^G^	nd	0.086 ± 0.004	nd	0.115 ± 0.004	nd	-
9 ^B^	nd	nd	1.19 ± 0.02	nd	30.88 ± 1.17	<0.001
10 ^B^	nd	nd	nd	nd	3.66 ± 0.01	-
11 ^D^	nd	0.215 ± 0.004	nd	nd	nd	-
12 ^B^	nd	nd	nd	nd	2.07 ± 0.06	-
13 ^H^	nd	1.016 ± 0.003 ^a^	nd	0.606 ± 0.002 ^b^	0.442 ± 0.001 ^c^	-
14 ^A^	0.1303 ± 0.002	nd	0.15 ± 0.01	nd	nd	0.001
15 ^I^	nd	nd	nd	nd	1.65 ± 0.03	-
16 ^E^	nd	1.07 ± 0.03	nd	nd	nd	-
17 ^I^	nd	nd	nd	nd	1.98 ± 0.76	-
18 ^A^	2.69 ± 0.05	nd	nd	0.263 ± 0.004	nd	<0.001
19 ^A^	0.82 ± 0.01	nd	nd	0.49 ± 0.02	nd	<0.001
20 ^J^	nd	tr	nd	nd	nd	-
21 ^J^	nd	nd	2.47 ± 0.04	2.27 ± 0.08	nd	<0.001
22 ^K^	2.33 ± 0.01	nd	nd	1.29 ± 0.01	nd	<0.001
23 ^K^	nd	nd	0.747 ± 0.004	nd	nd	-
24 ^G^	nd	nd	6.49 ± 0.23	5.77 ± 0.12	nd	<0.001
25 ^J^	nd	1.52 ± 0.03	nd	nd	0.90 ± 0.01	<0.001
26 ^L^	nd	4.58 ± 0.18	nd	nd	nd	-
27 ^G^	nd	nd	nd	1.996 ± 0.003	3.17 ± 0.07	-
28 ^J^	nd	nd	nd	1.19 ± 0.01	nd	-
29 ^K^	nd	1.99 ± 0.01	nd	nd	nd	-
30 ^J^	0.23 ± 0.003	nd	nd	1.59 ± 0.01	nd	-
31 ^J^	nd	nd	nd	3.533 ± 0.003	nd	-
32 ^J^	nd	nd	5.91 ± 0.02	nd	nd	-
33 ^J^	nd	nd	nd	nd	2.29 ± 0.01	-
34 ^L^	nd	8.73 ± 0.11 ^d^	7.05 ± 0.13 ^c^	10.08 ± 0.22 ^b^	15.92 ± 0.34 ^a^	-
35 ^L^	nd	3.17 ± 0.04	nd	nd	8.09 ± 0.25	<0.001
36 ^K^	0.61 ± 0.01	nd	nd	nd	nd	-
37 ^F^	0.107 ± 0.004	nd	nd	nd	nd	-
38 ^K^	nd	6.12 ± 0.12	1.23 ± 0.02	nd	nd	<0.001
39 ^K^	0.828 ± 0.013 ^c^	nd	nd	1.84 ± 0.05 ^a^	1.55 ± 0.07 ^b^	-
40 ^K^	nd	nd	1.71 ± 0.07	1.79 ± 0.01	nd	<0.001
41 ^J^	1.19 ± 0.05	nd	nd	nd	nd	-
42 ^K^	10.84 ± 0.14 ^d^	20.78 ± 0.34 ^b^	22.65 ± 0.33 ^a^	20.66 ± 0.26 ^b^	19.9 ± 0.13 ^c^	-
43 ^K^	nd	9.60 ± 0.39	nd	nd	nd	-
44 ^J^	nd	nd	0.278 ± 0.005	nd	nd	-
45 ^D^	nd	nd	3.16 ± 0.02	nd	nd	-
46 ^J^	nd	nd	1.19 ± 0.04	0.88 ± 0.03	nd	<0.001
47 ^J^	nd	0.68 ± 0.01	nd	nd	nd	-
48 ^K^	nd	nd	3.34 ± 0.02	5.46 ± 0.19	nd	-
49 ^K^	6.84 ± 0.09 ^a^	nd	nd	1.14 ± 0.03 ^c^	5.61 ± 0.09 ^b^	-
50 ^M^	nd	nd	1.03 ± 0.01	nd	nd	-
51 ^J^	nd	nd	nd	0.40 ± 0.02	nd	-
52 ^G^	nd	nd	36.04 ± 0.05	nd	nd	-
53 ^K^	nd	nd	nd	0.95 ± 0.01	nd	-
54 ^K^	2.59 ± 0.03	nd	nd	nd	0.87 ± 0.01	<0.001
55 ^J^	nd	nd	nd	0.080 ± 0.001	nd	-
56	nd	nd	nq	nd	nd	-
57	nd	nd	nd	nd	nq	-
58	nd	nd	nq	nd	nd	-
59 ^G^	nd	nd	0.026 ± 0.001	nd	nd	-
60 ^K^	nd	nd	1.07 ± 0.06	nd	nd	-
61 ^K^	nd	nd	0.25 ± 0.01	nd	nd	-
62 ^K^	nd	nd	7.28 ± 0.13	nd	nd	-
63 ^K^	nd	nd	9.22 ± 0.21	nd	nd	-
TPA	28.51 ± 0.28 ^e^	40.59 ± 0.13 ^b^	31.73 ± 0.23 ^d^	34.34 ± 0.10 ^c^	68.30 ± 0.78 ^a^	-
TiF	nd	nd	17.81 ± 0.14	nd	nd	-
TFa	nd	0.086 ± 0.004 ^d^	6.495 ± 0.23 ^b^	7.88 ± 0.12 ^a^	3.17 ± 0.07 ^c^	-
TFo	1.42 ± 0.05 ^e^	19.7 ± 0.3 ^d^	53.98 ± 0.08 ^a^	20.58 ± 0.19 ^c^	27.66 ± 0.08 ^b^	-
TF3O	nd	nd	nd	nd	3.64 ± 0.04	-
TOC	nd	0.215 ± 0.004	3.16 ± 0.02	nd	nd	<0.001
TPC	29.93 ± 0.34 ^e^	60.58 ± 0.45 ^d^	113.19 ± 0.68 ^a^	62.79 ± 0.41 ^c^	102.78 ± 0.84 ^b^	-

nd—not detected; tr—traces; nq—non quantifiable. TPA—Total Phenolic acids; TiF—Total isoflavones; TFa—Total flavanones: TFo—Total flavonoids; TF3O—Total flavan-3-ol; TOC—Total other compounds; TPC—Total phenolic compounds. Standard calibration curves used for quantification in each peak: A—caffeic acid; B—chlorogenic acid; C—syringic acid; D—cinnamic; E—*p*-coumaric acid; F—ferulic acid; G—naringenin; H—apigenin-6-*C*-glucoside; I—catequin; J—quercetin-3-*O*-rutinoside; K—rosmarinic acid; L—quercetin-3-*O*-glucoside; and M—apigenin-7-*O*-glucoside. ANOVA analysis—In each row different letters mean significant differences (*p* < 0.05).

**Table 4:** Characterization of organic acids and tocopherols in mixtures of dried tisanes (extracts) and infusions preparations (mean ± SD).

	Extracts					Infusion Preparations				
	T1	T2	T3	T4	T5	T1	T2	T3	T4	T5
Organic acids	(g/100 g·dw)					(mg/100 mL)				
Oxalic acid	2.47 ± 0.01 ^a^	0.38 ± 0.002 ^d^	1.30 ± 0.02 ^b^	0.79 ± 0.01 ^c^	tr	98 ± 1 ^a^	6.9 ± 0.1 ^d^	16.8 ± 0.3 ^c^	21.1 ± 0.5 ^b^	tr
Quinic acid	1.65 ± 0.07 ^a^	0.10 ± 0.04 ^d^	1.00 ± 0.08 ^c^	1.33 ± 0.09 ^b^	nd	69 ± 1 ^a^	2.8 ± 0.1 ^d^	28 ± 1 ^c^	33 ± 1 ^b^	nd
Malic acid	0.48 ± 0.03 ^a^	0.36 ± 0.02 ^d^	0.41 ± 0.01 ^c^	0.43 ± 0.01 ^b^	tr	68 ± 1 ^a^	11.5 ± 0.3 ^b^	11 ± 1 ^b^	10.5 ± 0.1 ^c^	tr
Fumaric acid	nd	nd	nd	nd	tr	nd	nd	nd	nd	tr
Sum	4.59 ± 0.09 ^a^	0.83 ± 0.07 ^d^	2.71 ± 0.08 ^b^	2.54 ± 0.09 ^c^	tr	234 ± 3 ^a^	21.2 ± 0.4 ^d^	56 ± 2 ^c^	65 ± 1 ^b^	tr
Tocopherols	(mg/100 g·dw)					(mg/100 mL)				
*α*-Tocopherol	12.2 ± 0.9 ^a^	3.3 ± 0.3 ^c^	0.87 ± 0.01 ^e^	3.5 ± 0.2 ^b^	2.3 ± 0.1 ^d^	nd	nd	nd	nd	nd
*γ*-Tocopherol	35.8 ± 0.4 ^b^	nd	4.71 ± 0.01 ^d^	11.2 ± 0.3 ^c^	109 ± 5 ^a^	nd	nd	nd	nd	nd
Sum	48.0 ± 0.6 ^b^	3.3 ± 0.3 ^e^	5.58 ± 0.01 ^d^	14.7 ± 0.3 ^c^	112 ± 5 ^a^	nd	nd	nd	nd	nd

dw—dry weight basis; nd—not detected; tr—traces. ANOVA analysis—In each row different letters mean significant differences (*p* < 0.05).

**Table 5:** Bioactive properties of the five tisanes prepared from five MAP mixtures (mean ± SD).

	T1		T2		T3		T4		T5	
Antioxidant Activity (IC_50_ values, μg mL) ^A^										
OxHLIA Δ*t* = 120	27.9 ± 0.2 ^a^		17.8 ± 0.2 ^e^		22.0 ± 0.2 ^d^		25.2 ± 0.3 ^b^		23.4 ± 0.3 ^c^	
Δ*t* = 180	45.3 ± 0.3 ^a^		30.3 ± 0.3 ^e^		36.5 ± 0.2 ^c^		37.3 ± 0.2 ^b^		34.4 ± 0.7 ^d^	
TBARS inhibition	13.2 ± 0.3 ^a^		7.4 ± 0.1 ^c^		9.7 ± 0.3 ^b^		9.8 ± 0.1 ^b^		5.07 ± 0.04 ^d^	
Antimicrobial activity (mg/mL) ^B^	MIC	MBC	MIC	MBC	MIC	MBC	MIC	MBC	MIC	MBC
Gram-negative bacteria										
*Escherichia coli*	20	>20	20	>20	>20	>20	>20	>20	20	>20
*Klebsiella pneumoniae*	20	>20	20	>20	>20	>20	>20	>20	>20	>20
*Morganella morganii*	>20	>20	20	>20	>20	>20	>20	>20	>20	>20
*Proteus mirabilis*	>20	>20	>20	>20	>20	>20	>20	>20	>20	>20
*Pseudomonas aeruginosa*	>20	>20	20	>20	>20	>20	>20	>20	>20	>20
Gram-positive bacteria										
*Enterococcus faecalis*	10	>20	20	>20	5	>20	10	>20	>20	>20
*Listeria monocytogenes*	5	>20	>20	>20	5	>20	20	>20	>20	>20
MRSA	10	>20	2.5	>20	10	>20	5	>20	10	>20
Anti-inflammatory activity (IC_50_ values µg/mL) ^C^										
RAW 264.7	>400		>400		>400		354 ± 6 *		345 ± 9 *	
Cytotoxicity Activity (GI_50_ values µg/mL) ^D^										
HepG2	311 ± 9 ^a^		>400		299 ± 9 ^b^		264 ± 15 ^c^		316 ± 13 ^a^	
NCI H460	257 ± 9 ^b^		>400		>400		248 ± 10 ^c^		317 ± 8 ^a^	
HeLa	294 ± 14 ^b^		>400		>400		253 ± 8 ^c^		313 ± 5 ^a^	
MCF7	320 ± 3 ^a^		>400		>400		308 ± 13 ^b^		285 ± 10 ^c^	
Hepatotoxicity (GI_50_ values µg/mL) ^D^										
PLP2	>400		>400		>400		>400		>400	
Anti-tyrosinase (IC_50_ values mg/mL or PI at 8 mg/mL) ^E^										
Mushroom tyrosinase inhibition	>8		>8		>8		>8		>8	
Antidiabetic activity (IC_50_ values µg/mL or PI at 8 mg/mL) ^F^										
Mammalian α-glucosidase inhibition	>8		>8		>8		>8		37 ± 6	
Yeast α-glucosidase inhibition	41% ± 4%		1.19 ± 0.02		43% ± 3%		6.9 ± 0.4		0.054 ± 0.003	

^A^ Trolox IC_50_ values: 5.8 ± 0,6 μg/mL (TBARS), 41 ± 3 μg/mL (OxHLIA 120 min) and 64 ± 2 μg/mL (OxHLIA 180 min); ^B^ Amoxicillin/Clavulanic Acid (E. Coli ≤ 8/4, S; *K. pneumoniae* ≤ 8/4, S; *K. pneumoniae* ESBL ≥ 32, R; M. morganii > 16/8, R); ^E^ Amikacin (*E. Coli* ESBL 16, I); ^F^ Vancomycin (*E. faecalis*, MRSA, and MSSA ≤ 2, S); ^G^ Ampicillin (*L. monocytogenes* ≤ 0.2, S). S—susceptible; I—intermediate; R—resistant. This classification was made according to the interpretative breakpoints suggested by Clinical and Laboratory Standards Institute (CLSI) and the European Committee on Antimicrobial Susceptibility Testing (EUCAST); ^C^ Dexamethaxone IC_50_ value: 16 ± 1 μg/mL; ^D^ Ellipticine GI50 values: 1.1 ± 0.2 μg/mL (HepG2), 1.03 ± 0.09 μg/mL (NCI-H460), 1.91 ± 0.06 μg/mL (HeLa), 0.91 ± 0.04 μg/mL (MCF-7), and 3.2 ± 0.7 μg/mL (PLP2); ^E^ Kojic acid IC_50_ values: 0.078 ± 0.001 μg/mL; ^F^ Acarbose IC_50_ values: 0.83 ± 0.02 μg/mL (yeast) and 0.86 ± 0.01 μg/mL (rat). In each row and for the different extraction procedure, different letters mean significant differences (*p* < 0.05). * *p*-value resulting from the Student *t* test: <0.001. MIC—minimum inhibitory concentration. MBC—minimum bactericidal concentration.
